# Extracellular vesicles highlight many cases of photoreceptor degeneration

**DOI:** 10.3389/fnmol.2023.1182573

**Published:** 2023-05-18

**Authors:** William J. Spencer

**Affiliations:** Department of Ophthalmology and Visual Sciences, SUNY Upstate Medical University, Syracuse, NY, United States

**Keywords:** photoreceptor degeneration, retina, outer segment, rhodopsin, ectosome, extracellular vesicle, microvesicle, cilia

## Abstract

The release of extracellular vesicles is observed across numerous cell types and serves a range of biological functions including intercellular communication and waste disposal. One cell type which stands out for its robust capacity to release extracellular vesicles is the vertebrate photoreceptor cell. For decades, the release of extracellular vesicles by photoreceptors has been documented in many different animal models of photoreceptor degeneration and, more recently, in wild type photoreceptors. Here, I review all studies describing extracellular vesicle release by photoreceptors and discuss the most unifying theme among them–a photoreceptor cell fully, or partially, diverts its light sensitive membrane material to extracellular vesicles when it has defects in the delivery or morphing of this material into the photoreceptor’s highly organized light sensing organelle. Because photoreceptors generate an enormous amount of light sensitive membrane every day, the diversion of this material to extracellular vesicles can cause a massive accumulation of these membranes within the retina. Little is known about the uptake of photoreceptor derived extracellular vesicles, although in some cases the retinal pigment epithelial cells, microglia, Müller glia, and/or photoreceptor cells themselves have been shown to phagocytize them.

## 1. Introduction

The release of small vesicles to the extracellular space is observed in cells from all domains of life and can serve a plethora of functions (Doyle and Wang, 2019; Gill et al., 2019). In cases of disease, extracellular vesicles (EVs) may play a beneficial role and/or promote pathology. For example, during Alzheimer’s disease neurons can protect themselves by discarding EVs packed with amyloid β fibrils to be phagocytized by microglia (Yuyama et al., 2012). Conversely, and also during Alzheimer’s disease, EVs can serve as the vehicle for neuron to neuron transfer of toxic intracellular Tau protein and promote pathology (Wang et al., 2017). The role that EVs play in retinal disease, and their potential application for retinal therapies, is a rapidly developing field of research (Kalargyrou et al., 2023). In this review, I focus on cases of EV release by photoreceptor cells and examine the mechanisms of vesicle release in these instances and their links to blinding retinal diseases.

### 1.1. Ectosomes versus exosomes

**FIGURE 1:**
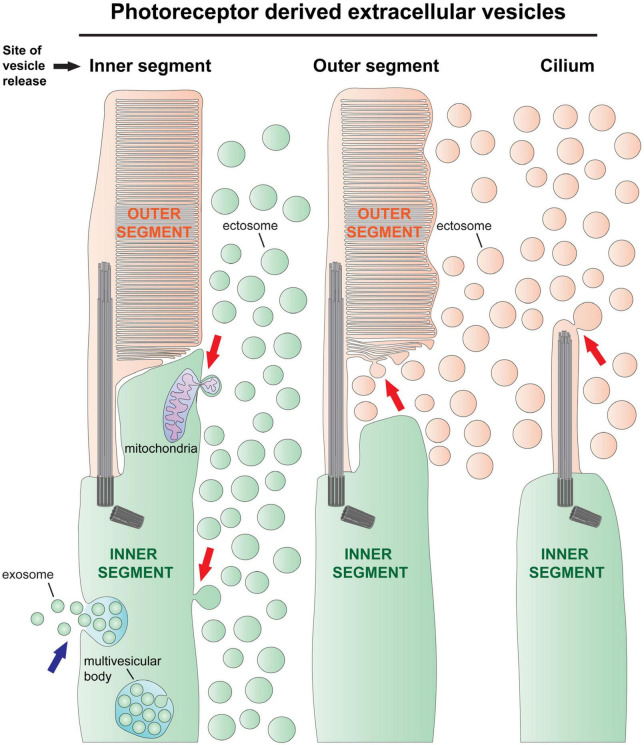
The types of extracellular vesicle release by photoreceptor cells. There are two types of extracellular vesicles–ectosomes and exosomes. Exosomes are formed by the fusion of a multivesicular body with the cell’s plasma membrane, releasing numerous exosomes together (blue arrow on left). Although there is evidence in support of exosome release by photoreceptors (Kalargyrou et al., 2021), this process has not been documented *in vivo*. Ectosomes originate by the outward budding of the cell’s plasma membrane, releasing a single vesicle directly to the extracellular space (red arrows). **Left**, ectosomes are often released from the photoreceptor’s inner segment plasma membrane [e.g., release driven by rhodopsin mislocalization (Li et al., 1996), see also Figure 2D], and sometimes contain pieces of mitochondria (Giarmarco et al., 2020). **Middle**, ectosomes have been observed budding from the newly forming disc membrane evaginations in *Prcd*^–/–^ mice (Spencer et al., 2019a) and in tunicamycin treated retinas (Fliesler et al., 1985; see also Figure 2C). **Right**, ectosomes are released from the ciliary plasma membrane of *rds* mice, which lack expression of peripherin-2 (Salinas et al., 2017; see also Figure 2A). In this mouse line, the photoreceptor cilia do not elaborate outer segments because all outer segment material is released in the form of ectosomes.

Based on their mode of formation, there are two types of EVs: exosomes and ectosomes (Cocucci and Meldolesi, 2015). *Exosomes* originate inside the cell as intraluminal vesicles that form by the inward budding and scission of an endosome’s membrane to form a multivesicular endosome (aka multivesicular body) (Figure 1). The endosomes can either fuse with lysosomes for degradation or fuse with the cell’s plasma membrane and release all their intraluminal vesicles extracellularly, at which point these vesicles would be called exosomes. In contrast to the exosome, an *ectosome*, also known as a microvesicle, is formed by the outward budding and scission of the cell’s plasma membrane, releasing the individual vesicle directly to the extracellular space (Figure 1).

In some cases, ectosomes can be distinguished from exosomes by size. Ectosomes vary from 100 to 1,000 nm in diameter while exosomes range between 50 and 200 nm in diameter (Cocucci and Meldolesi, 2015; Gurung et al., 2021). Because small ectosomes overlap in size with exosomes, diameter is only useful for identifying extracellular vesicles >200 nm as ectosomes and cannot be used to positively identify exosomes. Exosomes may be distinguished from ectosomes by the detection of exosome protein markers enriched in the vesicles. The most canonical markers include ALIX, a protein involved in the inward budding of the endosome membrane (Baietti et al., 2012) and the tetraspanins CD9, CD81, and CD63, which play a role in exosome cargo sorting (Andreu and Yanez-Mo, 2014; Suarez et al., 2021). Unlike for exosomes, ubiquitous ectosome-specific protein markers have yet to be elucidated, likely reflecting the huge diversity in ectosome composition, even among ectosomes released by the same cells (Kowal et al., 2016).

The molecular machinery involved in exosome formation has been well studied (Cocucci and Meldolesi, 2015). A cornerstone of this machinery is the ESCRT complex, which is unique in its ability to perform the scission of membranes that are budding in a direction away from the cytosol (Hurley, 2008). Exosomes fit this criteria–they begin as membrane buds toward the lumen of an endosome. The molecular machinery driving ectosome release is less understood. However, ectosome release also requires the scission of the membrane which has protruded in a direction away from the cytosol and, in some cases, ESCRT has been shown to be involved in ectosome release (Nabhan et al., 2012; Jackson et al., 2017; Xu et al., 2017). In other cases, ectosome formation appears independent of ESCRT (Nishimura et al., 2021; De Poret et al., 2022), perhaps reflecting that mechanisms of ectosome release are more diverse (Rilla, 2021). This is consistent with the fact that ectosomes can be widely different sizes, have diverse protein composition and may be shed from specialized structures along the plasma membrane, such as microvilli (Hurbain et al., 2022) and filipodia (Nishimura et al., 2021).

Another specialized plasma membrane structure, the primary cilium, has attracted significant interest for its ability to release ectosomes [recent reviews: (Wang and Barr, 2018; Luxmi and King, 2022; Ojeda Naharros and Nachury, 2022; Vinay and Belleannee, 2022)]. Some of the functions ascribed to ciliary ectosomes include selectively discarding proteins from the cilium (Nager et al., 2017), facilitating cell cycle reentry (Phua et al., 2017), and releasing bioactive proteins and peptides (Wood et al., 2013; Wang et al., 2014; Luxmi et al., 2022; Walsh et al., 2022). Although precise molecular mechanisms driving ciliary ectosome release are unknown, the process has been shown to rely on the actin cytoskeleton in cultured cells (Nager et al., 2017; Phua et al., 2017) and ESCRT in Chlamydomonas (Long et al., 2016).

## 2. Peripherin-2 blocks ciliary ectosome release to allow formation of the outer segment

Photoreceptor cells of the vertebrate retina are responsible for initiating vision by absorbing light and generating an electrical response that is relayed to the brain. To accomplish this, photoreceptors have specialized light sensing antennas called outer segments (recent reviews: Goldberg et al., 2016; Spencer W. J. et al., 2020; Wensel et al., 2021). These organelles are gigantic, highly modified primary cilia packed with hundreds of disc-shaped membrane layers called “discs,” that house photopigment (e.g., rhodopsin) and other proteins required to generate an electrical response to light. Photoreceptor outer segments are constantly renewed with dozens of new discs built at the base and dozens of old discs shed from the tip every day (Young, 1967).

**FIGURE 2:**
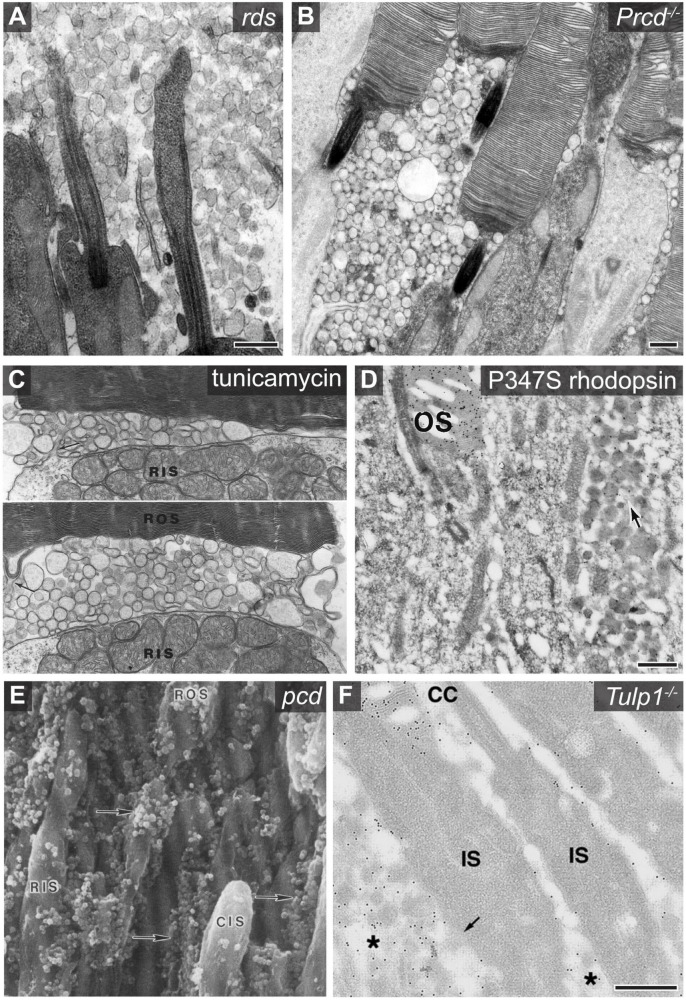
Examples of photoreceptor derived extracellular vesicle accumulation associated with retinal pathology. **(A)** An electron micrograph showing the accumulation of photoreceptor derived ciliary ectosomes in the subretinal space of *rds* mice. Scale bar is 500 nm. Image used with permission of Rockefeller University Press, from Salinas et al. (2017); permission conveyed through Copyright Clearance Center, Inc. **(B)** An electron micrograph showing the accumulation of ectosomes between photoreceptor cells of *Prcd*^–/–^ mice. Scale bar is 500 nm. Image adapted from Spencer et al. (2019a). Copyright 2019 National Academy of Sciences. **(C)** Two electron micrographs centered at the base of *Xenopus* photoreceptor outer segments showing the accumulation of vesicles between the rod inner segment (RIS) and rod outer segment (ROS) after a 6 h incubation of the retina with tunicamycin. Protrusions of the inner segment plasma membrane (arrow in top image) and most basal disc membrane (arrow in bottom image) indicate possible sites of origin for the vesicles. Image adapted from Wetzel et al. (1993). **(D)** An electron micrograph of an anti-rhodopsin immunogold labeled retinal cross section obtained from a transgenic mouse expressing P347S rhodopsin. The arrow denotes the accumulation of rhodopsin containing extracellular vesicles between photoreceptor cells. Scale bar is 500 nm. The outer segment (OS) is labeled. Image adapted from Li et al. (1996). Copyright 1996 National Academy of Sciences. **(E)** A scanning electron micrograph of a *pcd* mouse retinal cross section which shows the accumulation of extracellular vesicles adjacent to photoreceptor inner and outer segments. The vesicles, highlighted with arrows, were originally described as spherical excrescences or spherules based on their appearance. Image reprinted from Blanks et al. (1982), with permission from Elsevier. **(F)** An electron micrograph of an anti-rhodopsin immunogold labeled retinal cross section obtained from a *Tulp1*^–/–^ mouse retina. The asterisk labels extracellular vesicles which are enriched with rhodopsin. The outer segment (OS), connecting cilium (CC), and inner segment (IS) are labeled. Scale bar is 500 nm. Image used with permission from Association for Research in Vision and Ophthalmology, from Hagstrom et al. (2001); permission conveyed through Copyright Clearance Center, Inc.

It has long been known that the photoreceptors of *rds* mice, lacking expression of the photoreceptor specific protein peripherin-2 (Travis et al., 1991), do not form outer segments (Sanyal and Jansen, 1981), and degenerate (Sanyal et al., 1980). The photoreceptors from these mice were reported to completely lack disc membranes and form prototypic looking primary cilia which are surrounded by EVs (Figure 2A; Cohen, 1983; Jansen and Sanyal, 1984). The presence of rhodopsin in these vesicles confirmed that they originate from photoreceptor cells (Nir and Papermaster, 1986; Jansen et al., 1987; Usukura and Bok, 1987). More recently, Salinas et al. (2017) determined that the EVs accumulating in the subretinal space of *rds* mice are ciliary ectosomes and that the C-terminus of peripherin-2 functions to block ectosome release, thereby retaining this membrane material at the cilium so that it may be morphed into disc membranes (Figure 1). Evidence that the ectosomes exclusively originate from the cilium in *rds* mice comes from the fact that they lack Na/K-ATPase, a protein which is highly abundant in the photoreceptor’s plasma membrane but excluded from the cilium (Salinas et al., 2017). However, it was more recently shown that ectosomes originating from the photoreceptor’s inner segment plasma membrane can be enriched with mislocalized rhodopsin while lacking Na/K-ATPase (Ropelewski and Imanishi, 2020). This suggests that photoreceptors have an ectosome cargo sorting mechanism and that a fraction of the ectosomes accumulated in the subretinal space of *rds* mice could have originated from the inner segment plasma membrane.

## 3. Ectosome release from newly forming photoreceptor discs

While peripherin-2 is clearly necessary for suppressing ciliary ectosome release in photoreceptor cells, it is not sufficient. This is evident from the case of *Prcd*^–/–^ mice, which release ectosomes from the outer segment while having normal expression and localization of peripherin-2 (Figure 2B; Spencer et al., 2019a). Without PRCD, a defect in flattening of the newly forming discs causes the formation of swollen pockets in these membranes that are severed and released to the extracellular space as ectosomes (Figure 1). In contrast to *rds* mice whereby all disc membrane material is released in the form of ectosomes, the release of this material in *Prcd*^–/–^ mice is partial–most of the disc membrane material is properly flattened and incorporated inside the outer segment. This ultimately results in relatively normal looking photoreceptors with ectosomes accumulating near the inner/outer segment juncture and leads to the progressive death of photoreceptor cells (Allon et al., 2019; Spencer et al., 2019a). Decades prior to these studies of the *Prcd*^–/–^ mouse, Aguirre et al. (1982) made the first ever association of EVs with retinal degeneration while characterizing dogs affected by the disease from which *Prcd* is named, progressive rod-cone degeneration (Parkes et al., 1982; Aguirre and O’Brien, 1986). Like in the case of *Prcd*^–/–^ mice, EVs were observed surrounding degenerating outer segments in affected dog retinas. Genetic studies on dogs affected by this disease eventually led the same research group to discover the *Prcd* gene and determine that its mutation was the cause of retinal disease (Goldstein et al., 2006; Zangerl et al., 2006). PRCD is a 6 kDa, membrane anchored protein that specifically resides on the cytoplasmic surface of disc membranes where it interacts with rhodopsin and may help maintain rhodopsin packing density in discs (Skiba et al., 2013; Spencer et al., 2016; Sechrest et al., 2020). Despite recent progress, the molecular mechanism underlying PRCD’s role in disc formation and in preventing ectosome release remains to be elucidated.

Like *Prcd*^–/–^ mice, EVs have been observed surrounding newly forming discs in retinas exposed to the inhibitor of N-linked glycosylation, tunicamycin (Figure 2C; Fliesler et al., 1985; Defoe et al., 1986; Ulshafer et al., 1986; Wetzel et al., 1993; Wetzel and Besharse, 1994). This drug is remarkably specific in its toxicity to photoreceptor cells and the only morphological defect in the retina, prior to widespread degeneration, is the appearance of EVs at the base of outer segments (Fliesler et al., 1984; Spencer B. et al., 2020). Qualifying these vesicles as ectosomes, electron microscopy studies suggested that the EVs arose from the blebbing of the most basal, newly forming discs (Fliesler et al., 1985; Defoe et al., 1986; Ulshafer et al., 1986) and, potentially, the apical inner segment plasma membrane (Wetzel et al., 1993). The cause of ectosome formation induced by tunicamycin is unclear, although it may involve rhodopsin which is by far the most abundant glycoprotein in the outer segment and has two sites of N-linked glycosylation at its N-terminus which are completely blocked by tunicamycin without affecting the protein’s expression, folding, or trafficking (Fliesler et al., 1984; Kaushal et al., 1994). One untested hypothesis is that there is a loss in binding between two rhodopsin molecules present on apposing surfaces of newly forming discs, thereby disrupting membrane to membrane adhesion of discs and causing their vesiculation into the extracellular space (Fliesler and Basinger, 1985; Fliesler et al., 1985, 1986; Hubbell et al., 2003; Murray et al., 2009). The presence of swollen disc membranes in transgenic mice expressing mutant, non-glycosylated rhodopsin supports the idea of rhodopsin adhering disc membranes together (Murray et al., 2015). However, the fact that extracellular vesicles were not observed in these mice suggests tunicamycin may induce ectosome release in photoreceptors through a more complex mechanism involving a different glycosylated protein(s) and/or requiring the specific architecture of frog outer segments.

## 4. Rhodopsin mislocalization leads to massive ectosome release from photoreceptor inner segments

In addition to the outer segment or ciliary membrane, the inner segment plasma membrane is often the site of ectosome release from photoreceptor cells (Figure 1) and a common theme among these cases is the mislocalization of rhodopsin caused by mutations in its C terminus. Rhodopsin’s C terminus harbors its specific ciliary targeting sequence (Deretic et al., 1998) but is dispensable for its function as a photopigment (Weiss et al., 1994). Transgenic mice expressing mutant P347S rhodopsin were found to have a massive accumulation of EVs between photoreceptors, but otherwise, have nearly normal looking retinas (Li et al., 1996). While the bulk of rhodopsin, including the P347S mutant, was found to localize normally to the outer segment, a fraction is misdirected into EVs (Figure 2D). Other rhodopsin mutant animal models with this phenotype include rhodopsin P347L transgenic rabbits (Kondo et al., 2009; Muraoka et al., 2012), Q344ter transgenic mice (Concepcion and Chen, 2010), Ter349Glu knock-in mice (Hollingsworth and Gross, 2013), S334ter transgenic rats (LaVail et al., 2018; Yamauchi et al., 2018), and Q344ter transgenic frogs (Lodowski et al., 2013; Ropelewski and Imanishi, 2020). All these rhodopsin mutations result in a defect in the protein’s post-golgi trafficking to the cilium without affecting its processing in biosynthetic membranes, delivery to the plasma membrane, light absorption or other biochemical properties (Sung et al., 1991; Concepcion et al., 2002; Hollingsworth and Gross, 2013). The mislocalization of rhodopsin causes progressive photoreceptor cell death and, in humans, leads to autosomal dominant retinitis pigmentosa and blindness (Athanasiou et al., 2018). The cause of photoreceptor cell death due to rhodopsin mislocalization is currently unknown, although studies in mice and frogs have shown that degeneration does not rely on rhodopsin activation—it continues in complete darkness, on the background of transducin knockout and with mutant rhodopsin that is unable to bind chromophore (Tam et al., 2006; Concepcion and Chen, 2010). It remains to be determined what role EV accumulation, the unifying morphological defect caused by these mislocalized mutants, plays in the progression of photoreceptor cell death.

## 5. Defects in ciliary maintenance often lead to ectosome release from photoreceptors

In addition to rhodopsin itself, defects in many other proteins lead to rhodopsin mislocalization and the shedding of ectosomes from the inner segment. The first discovered case was the Purkinje cell degeneration (*pcd*) mouse which was clearly shown by electron microscopy to shed ectosomes directly from the photoreceptor inner segment plasma membrane (Figure 2E; Blanks et al., 1982), that are enriched with rhodopsin (Blanks and Spee, 1992). The *pcd* mouse has a mutation in the *Agtpbp1* gene which codes for a protein called CCP1 (aka NNA1) (Fernandez-Gonzalez et al., 2002) that is a deglutamylase acting in many types of neurons (Turay et al., 2021), including photoreceptors, where it plays a role in ciliary maintenance by modifying microtubules in the connecting cilium (Bosch Grau et al., 2017; Hotta et al., 2023). Similarly, the mislocalization of rhodopsin and shedding of EVs from photoreceptors has been observed in mouse models having disruption of other proteins crucial for maintenance of the cilia, including IFT88 (Pazour et al., 2002), IFT172 (Gupta et al., 2018), BBS8 (Dilan et al., 2018), ARL13B (Dilan et al., 2019), CEP290 (Potter et al., 2021), TUBBY (Heckenlively et al., 1995), TULP1 (Hagstrom et al., 1999, 2001), and TMEM138 (Guo et al., 2022). It has not clearly been shown in these cases whether the vesicles originate from the ciliary or inner segment plasma membrane, although, in contrast to *rds* and *Prcd*^–/–^ mice, the striking accumulation of vesicles between inner segments rather than outer segments in most cases argues that they are primarily released from the inner segment. However, a degree of ciliary ectosome release is possible given that severe defects in ciliary maintenance typically result in widespread mislocalization of ciliary proteins, which may include those known to block ectosome release from the photoreceptor cilium, like peripherin-2 or PRCD. For example, it was recently shown that PRCD is partially mislocalized from the outer segment in *Tulp1*^–/–^ mice (Remez et al., 2020) which may lead to some level of ectosome release from newly forming disc membranes as observed in *Prcd*^–/–^ mice (Allon et al., 2019; Spencer et al., 2019a). However, the prominent mislocalization of rhodopsin in *Tulp1*^–/–^ mice (Hagstrom et al., 2001) is most likely driving the bulk of EV release observed in these mice because rhodopsin mislocalization is sufficient to induce massive ectosome release from the inner segment (Li et al., 1996; Lodowski et al., 2013) and the EVs are most concentrated around inner segments in *Tulp1*^–/–^ mice (Figure 2F; Hagstrom et al., 1999).

## 6. Release of extracellular vesicles by wild type photoreceptors

As described above, the massive release of rhodopsin containing ectosomes from the photoreceptor inner segment is a unifying theme among cases of photoreceptor degeneration marked by post-golgi trafficking defects in rhodopsin. Is the release of ectosomes a unique response to this specific pathology or is it a process happening normally in wild type photoreceptors that gets amplified during disease? Recently, Lewis et al. (2022) addressed this question by carefully looking at the inner segment plasma membrane of wild type photoreceptors by electron microscopy. They observed a small number of ectosomes adjacent to, and budding from, inner segments of wild type photoreceptor cells and showed that some of them contained rhodopsin. Further, they observed a few that appear to originate from mitochondria within the inner segment of wild type photoreceptors (Figure 1). A similar extrusion of mitochondrial material was recently shown in cones at night (Giarmarco et al., 2020) and in cones under stress (Hayes et al., 2021). The extracellular extrusion of mitochondrial material may function to remove damaged mitochondria or play a role in cell to cell signaling (Lyamzaev et al., 2022). The fact that ectosomes are released from the inner segment, with or without rhodopsin, in wild type photoreceptors is consistent with ectosome release being a ubiquitous, conserved cellular process that is amplified by mistargeted rhodopsin accumulating in the plasma membrane.

Another example of EV release from wild type photoreceptors was recently observed in primary cultures of photoreceptor cells purified from disassociated wild type mouse retinas (Kalargyrou et al., 2021). In these cultured cells, the presence of EVs adjacent to distinct plasma membrane protrusions is consistent with the vesicles being ectosomes, although when they were purified from the media, they were found to be enriched with LAMP1 (Kalargyrou et al., 2021), an exosome specific protein (Mathieu et al., 2021). The possibility of exosome release by photoreceptors is supported by the observation of multivesicular bodies in the inner segments of wild type photoreceptors (Figure 1; Hollyfield et al., 1985; Hollyfield and Rayborn, 1987; Kalargyrou et al., 2021). A challenge of future studies is to determine if photoreceptors release exosomes *in vivo*.

## 7. Clearance of photoreceptor-derived extracellular vesicles

Although relatively little is known, recent studies have investigated the fate of photoreceptor derived EVs in the retina. Lodowski et al. (2013) used a fluorescent protein photoconversion approach to approximate the turnover of inner segment derived ectosomes in frog photoreceptors expressing mutant rhodopsin. In this model, they found that most ectosomes are degraded within 6 days, and in a subsequent study, determined that they are phagocytized by the retinal pigment epithelium (RPE) (Ropelewski and Imanishi, 2020). The RPE was also shown to phagocytize ectosomes derived from outer segments in *Prcd*^–/–^ mice, although microglia, which migrate right to the site of vesicle release in this model, were observed having engulfed even more ectosomes than the RPE (Spencer et al., 2019a). Leveraging a Cre-loxP reporter system, Kalargyrou et al. (2021) showed that EVs purified from the media of photoreceptors in culture were specifically taken up by Müller glia cells when injected into the mouse retina. Most recently, wild type photoreceptor cells have been observed engulfing EVs (Lewis et al., 2022).

## 8. Conclusion

The release of EVs from photoreceptor cells is a widely observed phenomenon associated with numerous instances of photoreceptor degeneration. In nearly all cases, it is clear that the vesicles are ectosomes which formed by budding directly from the photoreceptor’s inner segment or ciliary plasma membrane. The molecular mechanism driving ectosome release in photoreceptors is unknown, although, as observed in other cases of ectosome release, it may involve the action of the ESCRT complex or the actin cytoskeleton. The latter is somewhat more intriguing because ectosomes released from photoreceptors have been found to be enriched with actin but lack ESCRT related proteins (Chaitin et al., 1988; Chaitin, 1991; Spencer et al., 2019b).

Although photoreceptor derived EV release occurs normally to some extent in healthy photoreceptor cells, it is massively amplified when photoreceptors are burdened with mistrafficked outer segment material or lack the ability to properly build their outer segments. While under this type of stress, the release of EVs from photoreceptors likely serves to discard unwanted protein material rather than functioning as a specific means of intercellular communication. In many cases, the abnormal accumulation of vesicles between photoreceptor cells is the only morphological defect observed prior to widespread retinal degeneration, suggesting that the EVs may contribute to retinal disease. EVs are often known to be toxic—they have been shown to promote a wide range of diseases, including fatty liver (Ipsen and Tveden-Nyborg, 2021), Alzheimer’s (Ruan et al., 2021), Parkinson’s (Emmanouilidou et al., 2010), and autoimmunity (Buzas et al., 2014). On the other hand, and demonstrating that EVs can be protective toward photoreceptor cells, exosomes released by the RPE or microglia have recently been shown to slow photoreceptor degeneration (Xu et al., 2019; Wang et al., 2021). Could photoreceptor derived EV release be protective? It is plausible that photoreceptors releasing unwanted protein via EVs could reduce proteotoxic stress, which is known to underlie many types of photoreceptor degeneration, including those with ectosome release (Lobanova et al., 2013). Given that photoreceptor derived EVs underlie numerous cases of retinal degeneration, future studies are needed to determine their pathobiological significance and the precise molecular mechanisms responsible for their formation and clearance–first steps toward developing novel therapies for these retinal diseases.

## Author contributions

The author confirms being the sole contributor of this work and has approved it for publication.

## References

[B1] Aguirre G. Alligood J. O’Brien P. Buyukmihci N. (1982). Pathogenesis of progressive rod-cone degeneration in miniature poodles. *Invest. Ophthalmol. Vis. Sci.* 23 610–630. 6215376

[B2] Aguirre G. O’Brien P. (1986). Morphological and biochemical studies of canine progressive rod-cone degeneration. 3H-fucose autoradiography. *Invest. Ophthalmol. Vis. Sci.* 27 635–655. 3700016

[B3] Allon G. Mann I. Remez L. Sehn E. Rizel L. Nevet M. J. (2019). PRCD is concentrated at the base of photoreceptor outer segments and is involved in outer segment disc formation. *Hum. Mol. Genet.* 28 4078–4088. 10.1093/hmg/ddz248 31628458

[B4] Andreu Z. Yanez-Mo M. (2014). Tetraspanins in extracellular vesicle formation and function. *Front. Immunol.* 5:442. 10.3389/fimmu.2014.00442 25278937 PMC4165315

[B5] Athanasiou D. Aguila M. Bellingham J. Li W. Mcculley C. Reeves P. J. (2018). The molecular and cellular basis of rhodopsin retinitis pigmentosa reveals potential strategies for therapy. *Prog. Retin. Eye Res.* 62 1–23. 10.1016/j.preteyeres.2017.10.002 29042326 PMC5779616

[B6] Baietti M. F. Zhang Z. Mortier E. Melchior A. Degeest G. Geeraerts A. (2012). Syndecan-syntenin-ALIX regulates the biogenesis of exosomes. *Nat. Cell Biol.* 14 677–685. 22660413 10.1038/ncb2502

[B7] Blanks J. C. Spee C. (1992). Retinal degeneration in the pcd/pcd mutant mouse: Accumulation of spherules in the interphotoreceptor space. *Exp. Eye Res.* 54 637–644. 10.1016/0014-4835(92)90019-o 1623950

[B8] Blanks J. C. Mullen R. J. Lavail M. M. (1982). Retinal degeneration in the pcd cerebellar mutant mouse. II. Electron microscopic analysis. *J. Comp. Neurol.* 212 231–246. 10.1002/cne.902120303 7153375

[B9] Bosch Grau M. Masson C. Gadadhar S. Rocha C. Tort O. Marques Sousa P. (2017). Alterations in the balance of tubulin glycylation and glutamylation in photoreceptors leads to retinal degeneration. *J. Cell Sci.* 130 938–949. 28104815 10.1242/jcs.199091

[B10] Buzas E. I. Gyorgy B. Nagy G. Falus A. Gay S. (2014). Emerging role of extracellular vesicles in inflammatory diseases. *Nat. Rev. Rheumatol.* 10 356–364. 24535546 10.1038/nrrheum.2014.19

[B11] Chaitin M. H. (1991). Actin filaments in the photoreceptor cilium of the rds mutant mouse. *Exp. Eye Res.* 53 107–113. 10.1016/0014-4835(91)90152-5 1879494

[B12] Chaitin M. H. Carlsen R. B. Samara G. J. (1988). Immunogold localization of actin in developing photoreceptor cilia of normal and rds mutant mice. *Exp. Eye Res.* 47 437–446. 10.1016/0014-4835(88)90054-1 3181327

[B13] Cocucci E. Meldolesi J. (2015). Ectosomes and exosomes: Shedding the confusion between extracellular vesicles. *Trends Cell Biol.* 25 364–372. 10.1016/j.tcb.2015.01.004 25683921

[B14] Cohen A. I. (1983). Some cytological and initial biochemical observations on photoreceptors in retinas of rds mice. *Invest. Ophthalmol. Vis. Sci.* 24 832–843. 6862791

[B15] Concepcion F. Chen J. (2010). Q344ter mutation causes mislocalization of rhodopsin molecules that are catalytically active: A mouse model of Q344ter-induced retinal degeneration. *PLoS One* 5:e10904. 10.1371/journal.pone.0010904 20532191 PMC2880002

[B16] Concepcion F. Mendez A. Chen J. (2002). The carboxyl-terminal domain is essential for rhodopsin transport in rod photoreceptors. *Vision Res.* 42 417–426. 10.1016/s0042-6989(01)00195-x 11853757

[B17] De Poret A. Dibsy R. Merida P. Trausch A. Inamdar K. Muriaux D. (2022). Extracellular vesicles containing the I-BAR protein IRSp53 are released from the cell plasma membrane in an Arp2/3 dependent manner. *Biol. Cell* 114 259–275. 10.1111/boc.202100095 35844059

[B18] Defoe D. M. Besharse J. C. Fliesler S. J. (1986). Tunicamycin-induced dysgenesis of retinal rod outer segment membranes. II. Quantitative freeze-fracture analysis. Invest. *Ophthalmol. Vis. Sci.* 27 1595–1601. 3771140

[B19] Deretic D. Schmerl S. Hargrave P. A. Arendt A. Mcdowell J. H. (1998). Regulation of sorting and post-Golgi trafficking of rhodopsin by its C-terminal sequence QVS(A)PA. *Proc. Natl. Acad. Sci. U.S.A.* 95 10620–10625. 10.1073/pnas.95.18.10620 9724753 PMC27944

[B20] Dilan T. L. Moye A. R. Salido E. M. Saravanan T. Kolandaivelu S. Goldberg A. F. X. (2019). ARL13B, a joubert syndrome-associated protein, is critical for retinogenesis and elaboration of mouse photoreceptor outer segments. *J. Neurosci.* 39 1347–1364. 10.1523/JNEUROSCI.1761-18.2018 30573647 PMC6381253

[B21] Dilan T. L. Singh R. K. Saravanan T. Moye A. Goldberg A. F. X. Stoilov P. (2018). Bardet-Biedl syndrome-8 (BBS8) protein is crucial for the development of outer segments in photoreceptor neurons. *Hum. Mol. Genet.* 27 283–294. 10.1093/hmg/ddx399 29126234 PMC5886228

[B22] Doyle L. M. Wang M. Z. (2019). Overview of extracellular vesicles, their origin, composition, purpose, and methods for exosome isolation and analysis. *Cells* 8:727. 10.3390/cells8070727 PMC6678302 31311206

[B23] Emmanouilidou E. Melachroinou K. Roumeliotis T. Garbis S. D. Ntzouni M. Margaritis L. H. (2010). Cell-produced alpha-synuclein is secreted in a calcium-dependent manner by exosomes and impacts neuronal survival. *J. Neurosci.* 30 6838–6851. 10.1523/JNEUROSCI.5699-09.2010 20484626 PMC3842464

[B24] Fernandez-Gonzalez A. La Spada A. R. Treadaway J. Higdon J. C. Harris B. S. Sidman R. L. (2002). Purkinje cell degeneration (pcd) phenotypes caused by mutations in the axotomy-induced gene, Nna1. *Science* 295 1904–1906. 10.1126/science.1068912 11884758

[B25] Fliesler S. J. Basinger S. F. (1985). Tunicamycin blocks the incorporation of opsin into retinal rod outer segment membranes. *Proc. Natl. Acad. Sci. U.S.A.* 82 1116–1120. 3156378 10.1073/pnas.82.4.1116 PMC397205

[B26] Fliesler S. J. Rapp L. M. Hollyfield J. G. (1984). Photoreceptor-specific degeneration caused by tunicamycin. *Nature* 311 575–577. 6332991 10.1038/311575a0

[B27] Fliesler S. J. Rayborn M. E. Hollyfield J. G. (1985). Membrane morphogenesis in retinal rod outer segments: Inhibition by tunicamycin. *J. Cell Biol.* 100 574–587. 3155750 10.1083/jcb.100.2.574 PMC2113453

[B28] Fliesler S. Rayborn M. Hollyfield J. Lark D. Normark S. Uhlin B. (1986). *Protein-carbohydrate interactions in biological systems*, ed. Lark D. L. (London: Academic Press), 191–205.

[B29] Giarmarco M. M. Brock D. C. Robbings B. M. Cleghorn W. M. Tsantilas K. A. Kuch K. C. (2020). Daily mitochondrial dynamics in cone photoreceptors. *Proc. Natl. Acad. Sci. U.S.A.* 117 28816–28827. 33144507 10.1073/pnas.2007827117 PMC7682359

[B30] Gill S. Catchpole R. Forterre P. (2019). Extracellular membrane vesicles in the three domains of life and beyond. *FEMS Microbiol. Rev.* 43 273–303. 10.1093/femsre/fuy042 30476045 PMC6524685

[B31] Goldberg A. F. Moritz O. L. Williams D. S. (2016). Molecular basis for photoreceptor outer segment architecture. *Prog. Retin. Eye Res.* 55 52–81. 27260426 10.1016/j.preteyeres.2016.05.003 PMC5112118

[B32] Goldstein O. Zangerl B. Pearce-Kelling S. Sidjanin D. J. Kijas J. W. Felix J. (2006). Linkage disequilibrium mapping in domestic dog breeds narrows the progressive rod-cone degeneration interval and identifies ancestral disease-transmitting chromosome. *Genomics* 88 541–550. 10.1016/j.ygeno.2006.05.013 16859891 PMC4006154

[B33] Guo D. Ru J. Xie L. Wu M. Su Y. Zhu S. (2022). Tmem138 is localized to the connecting cilium essential for rhodopsin localization and outer segment biogenesis. *Proc. Natl. Acad. Sci. U.S.A.* 119:e2109934119. 10.1073/pnas.2109934119 35394880 PMC9169668

[B34] Gupta P. R. Pendse N. Greenwald S. H. Leon M. Liu Q. Pierce E. A. (2018). Ift172 conditional knock-out mice exhibit rapid retinal degeneration and protein trafficking defects. *Hum. Mol. Genet.* 27 2012–2024. 10.1093/hmg/ddy109 29659833 PMC5961092

[B35] Gurung S. Perocheau D. Touramanidou L. Baruteau J. (2021). The exosome journey: From biogenesis to uptake and intracellular signalling. *Cell Commun. Signal* 19 47. 10.1186/s12964-021-00730-1 PMC8063428 33892745

[B36] Hagstrom S. A. Adamian M. Scimeca M. Pawlyk B. S. Yue G. Li T. (2001). A role for the Tubby-like protein 1 in rhodopsin transport. *Invest. Ophthalmol. Vis. Sci.* 42 1955–1962. 11481257

[B37] Hagstrom S. A. Duyao M. North M. A. Li T. (1999). Retinal degeneration in tulp1-/- mice: Vesicular accumulation in the interphotoreceptor matrix. *Invest. Ophthalmol. Vis. Sci.* 40 2795–2802. 10549638

[B38] Hayes M. J. Tracey-White D. Kam J. H. Powner M. B. Jeffery G. (2021). The 3D organisation of mitochondria in primate photoreceptors. *Sci. Rep.* 11:18863. 10.1038/s41598-021-98409-7 34552195 PMC8458444

[B39] Heckenlively J. R. Chang B. Erway L. C. Peng C. Hawes N. L. Hageman G. S. (1995). Mouse model for Usher syndrome: Linkage mapping suggests homology to Usher type I reported at human chromosome 11p15. *Proc. Natl. Acad. Sci. U.S.A.* 92 11100–11104. 10.1073/pnas.92.24.11100 7479945 PMC40579

[B40] Hollingsworth T. J. Gross A. K. (2013). The severe autosomal dominant retinitis pigmentosa rhodopsin mutant Ter349Glu mislocalizes and induces rapid rod cell death. *J. Biol. Chem.* 288 29047–29055. 10.1074/jbc.M113.495184 23940033 PMC3790004

[B41] Hollyfield J. G. Rayborn M. E. (1987). Endocytosis in the inner segment of rod photoreceptors: Analysis of *Xenopus laevis* retinas using horseradish peroxidase. *Exp. Eye Res.* 45 703–719. 10.1016/s0014-4835(87)80119-7 3428395

[B42] Hollyfield J. G. Varner H. H. Rayborn M. E. Liou G. I. Bridges C. D. (1985). Endocytosis and degradation of interstitial retinol-binding protein: Differential capabilities of cells that border the interphotoreceptor matrix. *J. Cell Biol.* 100 1676–1681. 10.1083/jcb.100.5.1676 4039328 PMC2113862

[B43] Hotta T. Plemmons A. Gebbie M. Ziehm T. A. Blasius T. L. Johnson C. (2023). Mechanistic analysis of CCP1 in generating DeltaC2 alpha-tubulin in mammalian cells and photoreceptor neurons. *Biomolecules* 13:357. 10.3390/biom13020357 36830726 PMC9952995

[B44] Hubbell W. L. Altenbach C. Hubbell C. M. Khorana H. G. (2003). Rhodopsin structure, dynamics, and activation: A perspective from crystallography, site-directed spin labeling, sulfhydryl reactivity, and disulfide cross-linking. *Adv. Protein Chem.* 63 243–290. 10.1016/s0065-3233(03)63010-x 12629973

[B45] Hurbain I. Mace A. S. Romao M. Prince E. Sengmanivong L. Ruel L. (2022). Microvilli-derived extracellular vesicles carry Hedgehog morphogenic signals for Drosophila wing imaginal disc development. *Curr. Biol.* 32 361–373.e6 10.1016/j.cub.2021.11.023 34890558

[B46] Hurley J. H. (2008). ESCRT complexes and the biogenesis of multivesicular bodies. *Curr. Opin. Cell Biol.* 20 4–11. 18222686 10.1016/j.ceb.2007.12.002 PMC2282067

[B47] Ipsen D. H. Tveden-Nyborg P. (2021). Extracellular vesicles as drivers of non-alcoholic fatty liver disease: Small particles with big impact. *Biomedicines* 9:93. 10.3390/biomedicines9010093 33477873 PMC7832840

[B48] Jackson C. E. Scruggs B. S. Schaffer J. E. Hanson P. I. (2017). Effects of inhibiting VPS4 support a general role for ESCRTs in extracellular vesicle biogenesis. *Biophys. J.* 113 1342–1352. 10.1016/j.bpj.2017.05.032 28629620 PMC5607042

[B49] Jansen H. G. Sanyal S. (1984). Development and degeneration of retina in RDS mutant mice: Electron microscopy. *J. Comp. Neurol.* 224 71–84. 6715580 10.1002/cne.902240107

[B50] Jansen H. G. Sanyal S. De Grip W. J. Schalken J. J. (1987). Development and degeneration of retina in RDS mutant mice: Ultraimmunohistochemical localization of opsin. *Exp. Eye Res.* 44 347–361. 2954840 10.1016/s0014-4835(87)80170-7

[B51] Kalargyrou A. A. Basche M. Hare A. West E. L. Smith A. J. Ali R. R. (2021). Nanotube-like processes facilitate material transfer between photoreceptors. *EMBO Rep.* 22 e53732. 10.15252/embr.202153732 34494703 PMC8567251

[B52] Kalargyrou A. A. Guilfoyle S. E. Smith A. J. Ali R. R. Pearson R. A. (2023). Extracellular vesicles in the retina - putative roles in physiology and disease. *Front. Mol. Neurosci.* 15:1042469. 10.3389/fnmol.2022.1042469 36710933 PMC9877344

[B53] Kaushal S. Ridge K. D. Khorana H. G. (1994). Structure and function in rhodopsin: The role of asparagine-linked glycosylation. *Proc. Natl. Acad. Sci. U.S.A.* 91 4024–4028. 8171029 10.1073/pnas.91.9.4024 PMC43715

[B54] Kondo M. Sakai T. Komeima K. Kurimoto Y. Ueno S. Nishizawa Y. (2009). Generation of a transgenic rabbit model of retinal degeneration. *Invest. Ophthalmol. Vis. Sci.* 50 1371–1377. 19074802 10.1167/iovs.08-2863

[B55] Kowal J. Arras G. Colombo M. Jouve M. Morath J. P. Primdal-Bengtson B. (2016). Proteomic comparison defines novel markers to characterize heterogeneous populations of extracellular vesicle subtypes. *Proc. Natl. Acad. Sci. U.S.A.* 113 E968–E977. 10.1073/pnas.1521230113 26858453 PMC4776515

[B56] LaVail M. M. Nishikawa S. Steinberg R. H. Naash M. I. Duncan J. L. Trautmann N. (2018). Phenotypic characterization of P23H and S334ter rhodopsin transgenic rat models of inherited retinal degeneration. *Exp. Eye Res.* 167 56–90. 10.1016/j.exer.2017.10.023 29122605 PMC5811379

[B57] Lewis T. R. Phan S. Kim K. Y. Jha I. Castillo C. M. Ding J. D. (2022). Microvesicle release from inner segments of healthy photoreceptors is a conserved phenomenon in mammalian species. *Dis. Model Mech.* 15:dmm049871. 10.1242/dmm.049871 PMC9796728 36420970

[B58] Li T. Snyder W. K. Olsson J. E. Dryja T. P. (1996). Transgenic mice carrying the dominant rhodopsin mutation P347S: Evidence for defective vectorial transport of rhodopsin to the outer segments. *Proc. Natl. Acad. Sci. U.S.A.* 93 14176–14181. 10.1073/pnas.93.24.14176 8943080 PMC19513

[B59] Lobanova E. S. Finkelstein S. Skiba N. P. Arshavsky V. Y. (2013). Proteasome overload is a common stress factor in multiple forms of inherited retinal degeneration. *Proc. Natl. Acad. Sci. U.S.A.* 110 9986–9991. 10.1073/pnas.1305521110 23716657 PMC3683722

[B60] Lodowski K. H. Lee R. Ropelewski P. Nemet I. Tian G. Imanishi Y. (2013). Signals governing the trafficking and mistrafficking of a ciliary GPCR, rhodopsin. *J. Neurosci.* 33 13621–13638. 10.1523/JNEUROSCI.1520-13.2013 23966685 PMC3755712

[B61] Long H. Zhang F. Xu N. Liu G. Diener D. R. Rosenbaum J. L. (2016). Comparative analysis of ciliary membranes and ectosomes. *Curr. Biol.* 26 3327–3335. 27866888 10.1016/j.cub.2016.09.055 PMC5173405

[B62] Luxmi R. King S. M. (2022). Cilia-derived vesicles: An ancient route for intercellular communication. *Semin. Cell Dev. Biol.* 129 82–92. 10.1016/j.semcdb.2022.03.014 35346578 PMC9378432

[B63] Luxmi R. Mains R. E. Eipper B. A. King S. M. (2022). Regulated processing and secretion of a peptide precursor in cilia. *Proc. Natl. Acad. Sci. U.S.A.* 119:e2206098119. 10.1073/pnas.2206098119 PMC9351486 35878031

[B64] Lyamzaev K. G. Zinovkin R. A. Chernyak B. V. (2022). Extrusion of mitochondria: Garbage clearance or cell-cell communication signals? *J. Cell. Physiol.* 237 2345–2356. 35253232 10.1002/jcp.30711

[B65] Mathieu M. Nevo N. Jouve M. Valenzuela J. I. Maurin M. Verweij F. J. (2021). Specificities of exosome versus small ectosome secretion revealed by live intracellular tracking of CD63 and CD9. *Nat. Commun.* 12:4389. 10.1038/s41467-021-24384-2 34282141 PMC8289845

[B66] Muraoka Y. Ikeda H. O. Nakano N. Hangai M. Toda Y. Okamoto-Furuta K. (2012). Real-time imaging of rabbit retina with retinal degeneration by using spectral-domain optical coherence tomography. *PLoS One* 7:e36135. 10.1371/journal.pone.0036135 22558356 PMC3338600

[B67] Murray A. R. Fliesler S. J. Al-Ubaidi M. R. (2009). Rhodopsin: The functional significance of asn-linked glycosylation and other post-translational modifications. *Ophthalmic Genet.* 30 109–120. 10.1080/13816810902962405 19941415 PMC2881540

[B68] Murray A. R. Vuong L. Brobst D. Fliesler S. J. Peachey N. S. Gorbatyuk M. S. (2015). Glycosylation of rhodopsin is necessary for its stability and incorporation into photoreceptor outer segment discs. *Hum. Mol. Genet.* 24 2709–2723. 10.1093/hmg/ddv031 25637522 PMC4406288

[B69] Nabhan J. F. Hu R. Oh R. S. Cohen S. N. Lu Q. (2012). Formation and release of arrestin domain-containing protein 1-mediated microvesicles (ARMMs) at plasma membrane by recruitment of TSG101 protein. *Proc. Natl. Acad. Sci. U.S.A.* 109 4146–4151. 10.1073/pnas.1200448109 22315426 PMC3306724

[B70] Nager A. R. Goldstein J. S. Herranz-Perez V. Portran D. Ye F. Garcia-Verdugo J. M. (2017). An actin network dispatches ciliary GPCRs into extracellular vesicles to modulate signaling. *Cell* 168 252–263.e14. 10.1016/j.cell.2016.11.036 28017328 PMC5235987

[B71] Nir I. Papermaster D. S. (1986). Immunocytochemical localization of opsin in the inner segment and ciliary plasma membrane of photoreceptors in retinas of rds mutant mice. *Invest. Ophthalmol. Vis. Sci.* 27 836–840. 2939037

[B72] Nishimura T. Oyama T. Hu H. T. Fujioka T. Hanawa-Suetsugu K. Ikeda K. (2021). Filopodium-derived vesicles produced by MIM enhance the migration of recipient cells. *Dev. Cell* 56 842–859.e8. 10.1016/j.devcel.2021.02.029 33756122

[B73] Ojeda Naharros I. Nachury M. V. (2022). Shedding of ciliary vesicles at a glance. *J. Cell Sci.* 135:jcs246553. 10.1242/jcs.246553 36222105 PMC10399986

[B74] Parkes J. H. Aguirre G. Rockey J. H. Liebman P. A. (1982). Progressive rod-cone degeneration in the dog: Characterization of the visual pigment. *Invest. Ophthalmol. Vis. Sci.* 23 674–678. 7129812

[B75] Pazour G. J. Baker S. A. Deane J. A. Cole D. G. Dickert B. L. Rosenbaum J. L. (2002). The intraflagellar transport protein, IFT88, is essential for vertebrate photoreceptor assembly and maintenance. *J. Cell Biol.* 157 103–113. 10.1083/jcb.200107108 11916979 PMC2173265

[B76] Phua S. C. Chiba S. Suzuki M. Su E. Roberson E. C. Pusapati G. V. (2017). Dynamic remodeling of membrane composition drives cell cycle through primary cilia excision. *Cell* 168 264–279.e15. 28086093 10.1016/j.cell.2016.12.032 PMC5660509

[B77] Potter V. L. Moye A. R. Robichaux M. A. Wensel T. G. (2021). Super-resolution microscopy reveals photoreceptor-specific subciliary location and function of ciliopathy-associated protein CEP290. *JCI Insight* 6:e145256. 10.1172/jci.insight.145256 34520396 PMC8564900

[B78] Remez L. Cohen B. Nevet M. J. Rizel L. Ben-Yosef T. (2020). TULP1 and TUB are required for specific localization of PRCD to photoreceptor outer segments. *Int. J. Mol. Sci.* 21:8677. 10.3390/ijms21228677 33213002 PMC7698587

[B79] Rilla K. (2021). Diverse plasma membrane protrusions act as platforms for extracellular vesicle shedding. *J. Extracell. Vesicles* 10:e12148. 10.1002/jev2.12148 34533887 PMC8448080

[B80] Ropelewski P. Imanishi Y. (2020). RPE cells engulf microvesicles secreted by degenerating rod photoreceptors. *eNeuro* 7 1–10. 10.1523/ENEURO.0507-19.2020 32376599 PMC7242815

[B81] Ruan Z. Pathak D. Venkatesan Kalavai S. Yoshii-Kitahara A. Muraoka S. Bhatt N. (2021). Alzheimer’s disease brain-derived extracellular vesicles spread tau pathology in interneurons. *Brain* 144 288–309. 33246331 10.1093/brain/awaa376 PMC7880668

[B82] Salinas R. Y. Pearring J. N. Ding J. D. Spencer W. J. Hao Y. Arshavsky V. Y. (2017). Photoreceptor discs form through peripherin-dependent suppression of ciliary ectosome release. *J. Cell Biol.* 216 1489–1499. 10.1083/jcb.201608081 28381413 PMC5412563

[B83] Sanyal S. Jansen H. G. (1981). Absence of receptor outer segments in the retina of RDS mutant mice. *Neurosci. Lett.* 21 23–26. 7207866 10.1016/0304-3940(81)90051-3

[B84] Sanyal S. De Ruiter A. Hawkins R. K. (1980). Development and degeneration of retina in rds mutant mice: Light microscopy. *J. Comp. Neurol.* 194 193–207. 7440795 10.1002/cne.901940110

[B85] Sechrest E. R. Murphy J. Senapati S. Goldberg A. F. X. Park P. S. Kolandaivelu S. (2020). Loss of PRCD alters number and packaging density of rhodopsin in rod photoreceptor disc membranes. *Sci. Rep.* 10:17885. 10.1038/s41598-020-74628-2 33087780 PMC7577997

[B86] Skiba N. P. Spencer W. J. Salinas R. Y. Lieu E. C. Thompson J. W. Arshavsky V. Y. (2013). Proteomic identification of unique photoreceptor disc components reveals the presence of PRCD, a protein linked to retinal degeneration. *J. Proteome Res.* 12 3010–3018. 10.1021/pr4003678 23672200 PMC3771658

[B87] Spencer B. Blumbergs P. Manavis J. Finnie J. (2020). Retinal photoreceptor damage produced in guinea pigs by tunicamycin. *Aust. Vet. J.* 98 424–428. 10.1111/avj.12987 32643145

[B88] Spencer W. J. Ding J. D. Lewis T. R. Yu C. Phan S. Pearring J. N. (2019a). PRCD is essential for high-fidelity photoreceptor disc formation. *Proc. Natl. Acad. Sci. U.S.A.* 116 13087–13096. 10.1073/pnas.1906421116 31189593 PMC6601265

[B89] Spencer W. J. Lewis T. R. Pearring J. N. Arshavsky V. Y. (2020). Photoreceptor discs: Built like ectosomes. *Trends Cell Biol.* 30 904–915. 10.1016/j.tcb.2020.08.005 32900570 PMC7584774

[B90] Spencer W. J. Lewis T. R. Phan S. Cady M. A. Serebrovskaya E. O. Schneider N. F. (2019b). Photoreceptor disc membranes are formed through an Arp2/3-dependent lamellipodium-like mechanism. *Proc. Natl. Acad. Sci. U.S.A.* 116 27043–27052. 10.1073/pnas.1913518117 31843915 PMC6936530

[B91] Spencer W. J. Pearring J. N. Salinas R. Y. Loiselle D. R. Skiba N. P. Arshavsky V. Y. (2016). Progressive Rod-Cone Degeneration (PRCD) Protein Requires N-Terminal S-Acylation and Rhodopsin Binding for Photoreceptor Outer Segment Localization and Maintaining Intracellular Stability. *Biochemistry* 55 5028–5037. 10.1021/acs.biochem.6b00489 27509380 PMC5513659

[B92] Suarez H. Andreu Z. Mazzeo C. Toribio V. Perez-Rivera A. E. Lopez-Martin S. (2021). CD9 inhibition reveals a functional connection of extracellular vesicle secretion with mitophagy in melanoma cells. *J. Extracell. Vesicles* 10:e12082. 10.1002/jev2.12082 34012515 PMC8114031

[B93] Sung C. H. Schneider B. G. Agarwal N. Papermaster D. S. Nathans J. (1991). Functional heterogeneity of mutant rhodopsins responsible for autosomal dominant retinitis pigmentosa. *Proc. Natl. Acad. Sci. U.S.A.* 88 8840–8844. 1924344 10.1073/pnas.88.19.8840 PMC52606

[B94] Tam B. M. Xie G. Oprian D. D. Moritz O. L. (2006). Mislocalized rhodopsin does not require activation to cause retinal degeneration and neurite outgrowth in *Xenopus laevis*. *J. Neurosci.* 26 203–209. 16399688 10.1523/JNEUROSCI.3849-05.2006 PMC6674333

[B95] Travis G. H. Sutcliffe J. G. Bok D. (1991). The retinal degeneration slow (RDS) gene product is a photoreceptor disc membrane-associated glycoprotein. *Neuron* 6 61–70. 10.1016/0896-6273(91)90122-g 1986774

[B96] Turay S. Eroz R. Basak A. N. (2021). A novel pathogenic variant in the 3’ end of the AGTPBP1 gene gives rise to neurodegeneration without cerebellar atrophy: An expansion of the disease phenotype? *Neurogenetics* 22 127–132. 10.1007/s10048-021-00643-8 33909173

[B97] Ulshafer R. J. Allen C. B. Fliesler S. J. (1986). Tunicamycin-induced dysgenesis of retinal rod outer segment membranes. I. A scanning electron microscopy study. Invest. *Ophthalmol. Vis. Sci.* 27 1587–1594. 3771139

[B98] Usukura J. Bok D. (1987). Changes in the localization and content of OPSIN during retinal development in the RDS mutant mouse: Immunocytochemistry and immunoassay. *Exp. Eye Res.* 45 501–515. 10.1016/s0014-4835(87)80061-1 2962880

[B99] Vinay L. Belleannee C. (2022). EV duty vehicles: Features and functions of ciliary extracellular vesicles. *Front. Genet.* 13:916233. 10.3389/fgene.2022.916233 36061180 PMC9438925

[B100] Walsh J. D. Wang J. Dehart M. Nikonorova I. A. Srinivasan J. Barr M. M. (2022). Tracking N- and C-termini of C. elegans polycystin-1 reveals their distinct targeting requirements and functions in cilia and extracellular vesicles. *PLoS Genet.* 18:e1010560. 10.1371/journal.pgen.1010560 36574451 PMC9829181

[B101] Wang J. Barr M. M. (2018). Cell-cell communication via ciliary extracellular vesicles: Clues from model systems. *Essays Biochem.* 62 205–213. 10.1042/EBC20170085 29717060 PMC6922582

[B102] Wang J. Silva M. Haas L. A. Morsci N. S. Nguyen K. C. Hall D. H. (2014). C. elegans ciliated sensory neurons release extracellular vesicles that function in animal communication. *Curr. Biol.* 24 519–525. 24530063 10.1016/j.cub.2014.01.002 PMC4659354

[B103] Wang Y. Balaji V. Kaniyappan S. Krüger L. Irsen S. Tepper K. (2017). The release and trans-synaptic transmission of Tau via exosomes. *Mol. Neurodegener.* 12:5. 10.1186/s13024-016-0143-y 28086931 PMC5237256

[B104] Wang Y. Zhang Q. Yang G. Wei Y. Li M. Du E. (2021). RPE-derived exosomes rescue the photoreceptors during retina degeneration: An intraocular approach to deliver exosomes into the subretinal space. *Drug Deliv.* 28 218–228. 10.1080/10717544.2020.1870584 33501868 PMC7850421

[B105] Weiss E. R. Osawa S. Shi W. Dickerson C. D. (1994). Effects of carboxyl-terminal truncation on the stability and G protein-coupling activity of bovine rhodopsin. *Biochemistry* 33 7587–7593. 10.1021/bi00190a011 8011624

[B106] Wensel T. G. Potter V. L. Moye A. Zhang Z. Robichaux M. A. (2021). Structure and dynamics of photoreceptor sensory cilia. *Pflugers Arch.* 473 1517–1537. 34050409 10.1007/s00424-021-02564-9 PMC11216635

[B107] Wetzel M. G. Besharse J. C. (1994). Transport of phosphatidylcholine to Xenopus photoreceptor rod outer segments in the presence of tunicamycin. *J. Neurocytol.* 23 333–342. 10.1007/BF01666523 8089706

[B108] Wetzel M. G. Bendala-Tufanisco E. Besharse J. C. (1993). Tunicamycin does not inhibit transport of phosphatidylinositol to Xenopus rod outer segments. *J. Neurocytol.* 22 397–412. 8315416 10.1007/BF01195560

[B109] Wood C. R. Huang K. Diener D. R. Rosenbaum J. L. (2013). The cilium secretes bioactive ectosomes. *Curr. Biol.* 23 906–911. 23623554 10.1016/j.cub.2013.04.019 PMC3850760

[B110] Xu B. Fu Y. Liu Y. Agvanian S. Wirka R. C. Baum R. (2017). The ESCRT-III pathway facilitates cardiomyocyte release of cBIN1-containing microparticles. *PLoS Biol.* 15:e2002354. 10.1371/journal.pbio.2002354 28806752 PMC5570487

[B111] Xu W. Wu Y. Hu Z. Sun L. Dou G. Zhang Z. (2019). Exosomes from microglia attenuate photoreceptor injury and neovascularization in an animal model of retinopathy of prematurity. *Mol. Ther. Nucleic Acids* 16 778–790. 10.1016/j.omtn.2019.04.029 31163320 PMC6545376

[B112] Yamauchi K. Tanabu R. Monai N. Gonome T. Xie Y. Takahashi S. (2018). The spectral-domain optical coherence tomography findings associated with the morphological and electrophysiological changes in a rat model of retinal degeneration, rhodopsin S334ter-4 rats. *BioMed Res. Int.* 2018:5174986. 10.1155/2018/5174986 30581855 PMC6276524

[B113] Young R. W. (1967). The renewal of photoreceptor cell outer segments. *J. Cell Biol.* 33 61–72. 6033942 10.1083/jcb.33.1.61 PMC2107286

[B114] Yuyama K. Sun H. Mitsutake S. Igarashi Y. (2012). Sphingolipid-modulated exosome secretion promotes clearance of amyloid-beta by microglia. *J. Biol. Chem.* 287 10977–10989. 10.1074/jbc.M111.324616 22303002 PMC3322859

[B115] Zangerl B. Goldstein O. Philp A. R. Lindauer S. J. Pearce-Kelling S. E. Mullins R. F. (2006). Identical mutation in a novel retinal gene causes progressive rod-cone degeneration in dogs and retinitis pigmentosa in humans. *Genomics* 88 551–563. 10.1016/j.ygeno.2006.07.007 16938425 PMC3989879

